# Molecular and Antigenic Properties of Mammalian Cell-Expressed *Theileria parva* Antigen Tp9

**DOI:** 10.3389/fimmu.2019.00897

**Published:** 2019-04-29

**Authors:** Reginaldo G. Bastos, Valentina Franceschi, Giulia Tebaldi, Timothy Connelley, W. Ivan Morrison, Donald P. Knowles, Gaetano Donofrio, Lindsay M. Fry

**Affiliations:** ^1^Department of Veterinary Microbiology and Pathology, Washington State University, Pullman, WA, United States; ^2^Department of Medical-Veterinary Science, University of Parma, Parma, Italy; ^3^Royal School of Veterinary Sciences, The Roslin Institute, University of Edinburgh, Edinburgh, United Kingdom; ^4^Animal Disease Research Unit, United States Department of Agriculture, Agricultural Research Service, Pullman, WA, United States

**Keywords:** *Theileria parva*, Tp9 antigen, antigenicity, vaccine, bovine

## Abstract

East Coast Fever (ECF), caused by the tick-borne apicomplexan parasite *Theileria parva*, is a leading cause of morbidity and mortality in cattle of sub-Saharan Africa. The infection and treatment method (ITM) is currently the only vaccine available to control *T. parva*. Although ITM elicits levels of protection, its widespread adoption is limited by costs, laborious production process, and antibiotic co-treatment requirement, necessitating the development of a more sustainable vaccine. To this end, efforts have been concentrated in the identification of new *T. parva* vaccine antigens and in the development of suitable platforms for antigen expression. In this study, we investigated the molecular and antigenic properties of *T. parva* antigen Tp9 expressed by mammalian cells. Data indicate that Tp9 contains a signal peptide that is weakly functional in mammalian cells. Thus, Tp9 secretion from mammalian cells increased 10-fold after the native signal peptide was replaced with the human tissue plasminogen activator signal peptide (tPA). Sera from all *T. parva*-immune cattle recognized this recombinant, secreted Tp9. Additionally, PBMC from ITM-immunized cattle produced significant (*p* < 0.05) amounts of IFNγ following *ex vivo* exposure to Tp9, but this response varied between cattle of different MHC class I and class II genotypes. In addition, depletion experiments demonstrated that IFNγ to Tp9 was primarily produced by CD4^+^ T cells. Molecular analysis demonstrated that Tp9 presents a signal peptide that is weakly functional in mammalian cells, suggesting that it remains within lymphocytes during infection. Tp9 secretion from mammalian cells was substantially increased when the tPA secretion signal sequence was substituted for the native secretion signal sequence. Using full-length, recombinant Tp9 secreted from mammalian cells, we demonstrated that *T. parva*-immune cattle develop both humoral and cellular immune responses to this antigen. Collectively, these results provide rationale for further evaluation of Tp9 as a component of a *T. parva* subunit vaccine.

## Introduction

*Theileria parva*, a tick-borne protozoan parasite of the phylum Apicomplexa and the causative agent of East Coast Fever (ECF), kills over a million cattle each year in Eastern, Central, and Southern Africa (1, 2). *T. parva* has a complex life cycle that involves the development of asexual stages in the bovine host and sexual stages in the tick vector, *Rhipicephalus appendiculatus*. *T. parva* sporozoites, present in infected tick salivary glands, are inoculated into cattle during tick feeding. At this point, sporozoites rapidly enter B and T lymphocytes, and develop into the schizont stage (3, 4). Schizonts induce neoplasia-like transformation of infected lymphocytes, and divide in concert with the transformed cells. Clonal expansion of infected cells, and the resultant immune response, leads to clinical signs of ECF, including lymphadenopathy, leukopenia, thrombocytopenia, fever, respiratory failure, and death (5).

Control of *T. parva* currently relies on extensive use of acaricides to limit tick infestation and on the infection and treatment method (ITM) of immunization, in which cattle are infected via subcutaneous inoculation of live *T. parva* sporozoite stabilate and co-treated with long-acting oxytetracycline. The use of acaricides has major drawbacks, including the development of resistant tick populations, food-safety concerns, and environmental contamination resulting from toxic residues (6). Although ITM elicits long-lived *T. parva* immunity, vaccine stabilate production is labor-intensive and expensive. Determination of sporozoite dose is difficult to standardize and requires large numbers of cattle to titrate each batch of vaccine. Vaccine costs are further increased by the requirement for oxytetracycline co-treatment, and these costs are often prohibitive to smallholder pastoralist farmers who are in greatest need of the vaccine (7, 8). In addition, ITM-immunized animals remain life-long, asymptomatic carriers of *T. parva*, which poses risk for the spread of the disease to non-immunized animals via tick bite (8, 9). Therefore, development of a next-generation vaccine to control *T. parva* is urgently needed.

It has been demonstrated that the protective immune response against *T. parva* requires development of a CD8^+^ T-cell response to schizont-infected lymphocytes (10–12). It has also been shown experimentally that induction of a robust antibody response to a recombinant sporozoite surface antigen, p67, can provide protection in a proportion of animals by preventing or reducing the entry of sporozoites into bovine lymphocytes (13–16). The immunogenic potential of another antigen, the polymorphic immunodominant molecule (PIM), has also been examined. PIM is expressed by both sporozoite and schizont stages of the parasite (17), but although it has been shown to induce both cellular and humoral immune responses, there is yet no evidence that it can stimulate immunity (16). An additional eight antigens, named Tp1-8, were identified as targets of MHC class I-restricted CD8^+^ T lymphocytes from *T. parva*-immune cattle (10). Subsequently, a further three antigens, named Tp9, Tp10, and Tp12, were also identified by screening CD8^+^ T cell lines from immune cattle (18, 19). Despite the fact that a subset of these antigens have been tested for immunogenicity (10), further studies are needed to fully evaluate their potential as vaccine candidates.

Concomitantly with the identification of novel antigens was extensive work to optimize antigen expression systems and delivery platforms for *Theileria* antigens. Both prokaryotic and eukaryotic systems have been tested for expression of potential *T. parva* vaccine antigens (15, 16, 20, 21). Codon usage, post-translational modifications, protein conformation, and solubility are a few aspects that have been considered in deciding the most suitable platform to express antigens for subunit vaccines. Recently, we examined the antigenicity of the full-length *T. parva* p67 protein expressed in a mammalian system, and that work provided a basis for further studies to investigate this recombinant, mammalian expressed antigen as a subunit vaccine component to prevent *T. parva* (21).

In the present study, we consider the hypothesis that a suitable platform to express *T. parva* vaccine antigens will maintain the antigenic properties of the native target protein. To this end, we expressed *Theileria parva* antigen Tp9 in a mammalian system and characterized its molecular and antigenic properties. We demonstrate that native Tp9 is poorly secreted from mammalian cells and likely remains within lymphocytes during infection. Its secretion was considerably augmented by replacing the native signal peptide by a canonical eukaryotic signal peptide. Using the recombinant, mammalian-expressed and secreted Tp9, we showed that *T. parva*-immune cattle develop both humoral and cellular immune responses to this antigen. We also developed several human and bovine-derived cell lines transduced by a third-generation lentiviral vector that constitutively express a stable and secreted Tp9 form that can be useful for diagnostic and immunization purposes. Collectively, these results establish a rationale for the use of the mammalian expressed Tp9 as a component of a subunit vaccine to control ECF.

## Materials and Methods

### Mammalian Cell Lines

Human Embryo Kidney (HEK) 293T (ATCC: CRL-11268), Madin Darby Canine Kidney Cells (MDCK, ATCC® CCL-34™) and Swine kidney epithelial cells (PK15, ATCC® CCL-33®) were maintained as suggested by the manufacturer's instructions. Equine Adipose-Derived Stromal Cells (EADSC), Bovine Bone Marrow Stromal Cells (BBMSC), and Alpaca derived Skin Stromal cells (ADSSC) were derived, immortalized, and maintained as previously described (22–24). All cell lines were cultured in Eagle's Minimal Essential Medium (EMEM, Gibco) containing 10% fetal bovine serum (FBS), 2 mM of L-glutamine (Gibco), 100 IU/mL of penicillin (Gibco), 100 μg/mL of streptomycin (SIGMA), and 0.25 μg/mL of amphotericin B (Gibco) and were incubated at 37°C/5% CO_2_ in a humidified incubator.

### Sequence Analysis and Cloning of Tp9

The codon-usage adapted Tp9 synthetic ORF was constructed from a published sequence (NCBI GenBank: JQ735949.1) (Supplementary Figure 1). In this synthetic construct, the natural predicted signal peptide was substituted with human tissue plasminogen activator signal peptide (tPA). In order to provide the natural Tp9 signal peptide (tp9SP), insert a *Nhe*I restriction site at the 5' terminus of the ORF, tag Tp9 ORF with the peptide epitope AU1 (DTYRYI) (25), and insert a *Sma*I restriction site at 3′ terminus, the tp9 ORF was amplified using PCR with a pTp9 DNA template and Tp9SP-Tp9 sense and Tp9 AU1 antisense primers (Supplementary Table 1). The PCR amplification reaction was performed in a final volume of 50 μl, containing 10 mM Tris–hydrochloride pH 8.3, 10% Dimethyl Sulfoxide (DMSO), 0.2 mM deoxy nucleotide triphosphates, 2.5 mM MgSO, 50 mM KCl, and 0.25 μM of each primer. pTP9 DNA was firstly linearized with *Pst*I to facilitate polymerase action and then amplified over 35 cycles, each cycle consisting of 1 min of denaturation at 94°C, 1 min of primer annealing at 58°C and 1 min of chain elongation with 1 U of *Pfu* DNA polymerase (Thermo Fisher Scientific) at 72°C. The amplicon was subsequently checked in 1% agarose gel and visualized after ethidium bromide staining in 1X TAE buffer (40 mM Tris-acetate, 1 mM EDTA).

pCMV-Tp9AU1 was generated by sub-cloning the amplified tp9sp-Tp9 ORF, cut with *Nhe*I/*Sma*I restriction enzymes, into the shuttle vector pINT2EGFP (26). It was then digested with the same enzymes to excise the EGFP ORF and put the Tp9 ORF under the transcriptional control of the strong Cytomegalovirus immediate early gene promoter and of the bovine growth hormone polyadenylation signal.

Full length Tp9 deprived of its signal peptide was obtained using PCR starting from the pTP9 DNA template using a Tp9 sense and Tp9 AU1 antisense primer pair (Supplementary Table 1) to insert a *Nhe*I restriction site at the 5′ terminus and a *Sma*I restriction site at the 3′ terminus with the AU1 tag. PCR amplification of this construct was performed as described above with the same parameters. The *Nhe*I/*Sma*I cut amplicon was cloned into the shuttle vector pINT2EGFP (26) and cut with the same enzymes to generate pCMV-ΔspTp9AU1.

The natural Tp9 signal peptide was also placed in front of the EGFP ORF through EGFP PCR amplification using the Tp9SP-GFP sense and GFP-AU1 antisense primer pair (Supplementary Table 1). pEGFPC1 DNA (Clontech) was firstly linearized with *Ase*I and then used as a template to amplify the EGFP ORF with the natural Tp9 signal peptide and a *Nhe*I restriction site at the 5' terminus and the AU1 tag and a *Xho*I restriction site at the 3' terminus. This amplicon was cut with *Nhe*I/*Xho*I and cloned into pEGFP-C1, in which the EGFP ORF was excised with the same restriction enzymes, to generate pCMV-Tp9spGFP.

Using PCR amplification, the tPA signal peptide was added to the Tp9 ORF (tPA-sp). Starting from the pTp9 DNA template, the *Sma*I-*Nhe*I Tp9 sense and Tp9 AU1 antisense primer pair (Supplementary Table 1) was used to insert the tPA signal peptide and *Nhe*I restriction site at the 5′ terminus and a *Sma*I restriction site at the 3′ terminus with an AU1 tag. The amplicon was restriction digested with *Nhe*I/*Sma*I and ligated into *Nhe*I/*Sma*I cut pINT2EGFP to generate pCMV-tPAspTp9AU1.

The lentiviral transfer vector pEF1α-tPA-Tp9-iresGFP, delivering full length tPA-Tp9AU1 with a tPA signal peptide was obtained via PCR amplification of tPAsp-Tp9 from pTP9 using *Sma*I-*Nhe*I Tp9 sense and Tp9 AU1 antisense primers (Supplementary Table 1). The PCR amplification reaction was carried out as described above. The amplicon was cut with *Sma*I and cloned into the *Pme*I linearized pWPI commercial vector (Addgene).

### Transient Transfection and Secretion of Tp9 From HEK 293T Cells

To evaluate the expression and secretion of Tp9, HEK 293T cells were transiently transfected with pCMV-Tp9AU1, pCMV-ΔspTp9AU1, pCMV-tPAspTp9AU1, pEF1α-tPA-Tp9-iresGFP, pCMV-Tp9spGFP, pEF1α-GFP (pWPI, Addgene), and pCMV-GFP (pEGFP-C1, negative control, Clontech) using Polyethylenimine (PEI) transfection reagent (Polysciences, Inc.). Briefly, cells were seeded at 3 × 10^5^ cells/well in 6-well plates and incubated overnight at 37°C/5% CO_2_ in a humidified incubator. Cells were then incubated for 6 h with a transfection mix containing 3 μg plasmid DNA and PEI (ratio 1:2.5 DNA-PEI) in Dulbecco's modified essential medium (DMEM) high glucose (Euroclone) without serum. After incubation, the transfection mix was replaced by fresh medium EMEM, with 10% FBS, 100 IU/ml of penicillin, 100 μg/ml of streptomycin and 0.25 μg/ml of amphotericin B, and incubated for 24 h at 37°C/5% CO_2_ in a humidified incubator. To test supernatant protein expression, the transfection solution was replaced with fresh DMEM/F12 (ratio 1:1) medium without FBS and incubated for 48 h at 37°C/5% CO_2_ in a humidified incubator. Cell supernatant was then collected and analyzed by immunoblot.

### Lentivirus Reconstitution and Transduction

HEK 293T cells were transfected in a T175 cm^2^ flask with 25 μg of pEF1α-tPA-Tp9-iresGFP transfer vector, 13 μg of p8.74 packaging vector, 10 μg of pMD2 pseudotyping vector, and 10 μg of pREV using PEI transfection reagent (Polysciences, Inc.). Briefly, 58 μg of DNA were mixed with 145 μg of PEI (1 mg/mL) (ratio 1:2.5 DNA-PEI) in 3 ml of Dulbecco's modified essential medium (DMEM) high glucose (Euroclone) without serum. After 15 min incubation at room temperature, 4x volumes of medium without serum were added and the transfection solution transferred to the cell monolayer and left for 6 h at 37°C/5% CO_2_, in a humidified incubator. The transfection mixture was then replaced with 25 ml of fresh medium EMEM supplemented with 10% FBS, 50 IU/ml of penicillin, 50 μg/ml of streptomycin and 2.5 μg/ml of amphotericin B and cells incubated for 48 h at 37°C/5% CO_2_. The flask was then stored at −80°C. Lentivirus was later obtained by subjecting cells to three freeze-thaw cycles, and then clarifying the supernatant via centrifugation at 3,500 rpm for 5 min at 4°C and filtering through a 0.45 μm filter (Millipore). The clarified supernatant was stored at −80°C. To obtain stably transduced cell lines, 1 × 10^5^ HEK 293T cells were infected with 2 × 10^5^ TU (transducing units) of reconstituted tPA-Tp9AU1 lentivirus. Cells were incubated overnight at 37°C/5% CO_2_ and the culture medium was then replaced with fresh medium supplemented with 10% of FBS. Transduced cells were observed daily via fluorescence microscopy for GFP expression to monitor the rate of transduction. ADSSC, BBMSC, EADSC, MDCK, and PK15 were similarly transduced. All the tPA-Tp9AU1 lentivirus transduced cell lines were detached, washed twice with sterile PBS, suspended in PBS, and subjected to analysis with a Tali image cytometer (Invitrogen) to assess transduction rate.

### Cattle, *T. parva* Infection, and Ethics Statements

This study was carried out in accordance with the recommendations of The U.S. Animal Welfare Act (United States Code, Title 7, Chapter 54, sections 2131–2159) and Animal Welfare Regulations (Code of Federal Regulations, Title 9, Chapter 1, Subchapter A, parts 1–4). The protocol was approved by the Washington State University Institutional Animal Care and Use Committee, protocol number 4980. Therapeutic drugs were administered according to the manufacturer's dosing instructions. Six MHC class I A10 or A14 haplotype-matched (27) Holstein-Friesian steer calves obtained at 3–6 months of age were utilized in this study. Pre-infection complete blood counts (CBCs) were normal, and all calves tested negative on a pre-infection *T. parva* PIM enzyme-linked immunosorbent assay (ELISA) as previously described (28). Four steers (#139, #147, #152, and #158) were infected with a *T. parva* Muguga stabilate and concomitantly treated with oxytetracycline as previously described (29). Beginning 3 days after ITM, the temperature, pulse, respiratory rate, and CBC were monitored daily for each animal. Steers that developed significant pyrexia (rectal temperature >40.2°C) were treated with flunixin meglumine, and those that developed dyspnea were treated with furosemide and buparvaquone. Animals were re-infected three more times at 6 month intervals, and were monitored for adverse reactions as above for each infection; however, no adverse clinical signs were detected during subsequent infections. Maintenance and boosting of *T. parva* immunity was verified by PIM ELISA. The remaining two steers (#141 and #1413) were used as uninfected negative controls. PBMC and serum samples from each steer were used for cellular and serological assays to investigate the antigenicity of Tp9.

### Immunoblot

Protein cell extracts were obtained from pCMV-Tp9AU1, pCMV-ΔspTp9AU1, pCMV-tPAspTp9AU1, pEF1α-tPA-Tp9-iresGFP, pCMV-Tp9spGFP, and pCMV-GFP transfected HEK 293T, or from tPA-Tp9AU1 lentivirus stably transduced ADSSC, BBMSC, EADSC, HEK 293T, MDCK and PK15 and un-transduced control cell lines by adding 100 μl of cell extraction buffer (50 mM Tris-HCl, 150 mM NaCl, and 1% NP-40; pH 8). After BCA total protein quantification (Pierce™ BCA Protein Assay kit, Thermo Fisher Scientific), cell extracts containing 5, 10, or 20 μg of total protein were electrophoresed through 10% SDS-PAGE. After that, proteins were transfer into nylon membranes by electroblotting, membranes were incubated with anti-AU1 rabbit polyclonal antibody (A190-125A, Bethyl laboratories Inc.) diluted 1:10.000, and then with a secondary antibody goat anti-rabbit immunoglobulin labeled with horse radish peroxidase (Sigma), diluted 1:15.000 to be visualized by enhanced chemiluminescence (Clarity Max Western ECL substrate, Bio-Rad). Also, cell supernatants, obtained from HEK 293T transfected with pCMV-Tp9AU1, pCMV-ΔspTp9AU1, pCMV-tPAspTp9AU1, pEF1α-tPA-Tp9-iresGFP, pCMV-Tp9spGFP, and pCMV-GFP and from all the transduced cell lines, were collected after 48 h in serum free medium DMEM-F12 secretion condition and analyzed through 10% SDS-PAGE gel electrophoresis and immunoblotting as described above.

### Tp9 ELISA

Serum samples from *T. parva*-immune and control cattle were used to evaluate the antigenicity of the mammalian expressed and secreted Tp9 by ELISA. Briefly, microplates (Immulon® Microtiter™, Thermo Fisher Scientific) were coated overnight at 4°C with 500 ng/well of HEK 293T cell supernatant containing Tp9 or GFP in 1x ELISA coating buffer (BioLegend). After blocking in 20% non-fat milk, serum samples diluted 1:100 were added to each well and the plate incubated for 1 h at room temperature. Mouse anti-AU1 monoclonal antibody (clone G8-D3, 1:200, Creative Diagnostics) was used as a control to verify binding of recombinant Tp9-AU1 to the ELISA plates. After 3 washes in 20% (v/v) Tween-20 in PBS (PBS-T), anti-bovine (1:5,000) (Jackson ImmunoResearch) or anti-mouse (1:5,000) (Abcam) IgG-HRP was added to each well, and the plates incubated for 1 h at room temperature. Plates were then washed three times in PBS-T and developed with TMB substrate (Thermo Fisher Scientific). The enzymatic reaction was stopped by adding 0.2M H_2_SO_4_ to each well, and the plates read at 450 nm using an ELISA plate reader (MultiSkan MCC, Thermo Fisher Scientific). Results of the Tp9 ELISA are presented as normalized OD450 where the OD of the wells containing supernatant from HEK 293T cells transfected with pEF1α-GFP was subtracted from the OD of the wells containing the Tp9 supernatant. Serum samples from *T. parva* infected and uninfected control animals were also tested for the presence of anti-PIM antibodies by ELISA as previously described (28).

### Generation of *T. parva*-Infected Lymphocyte Cell Lines

*T. parva* infected lymphocyte cell lines were established and maintained as previously described (30). Briefly, PBMC were isolated from whole blood using Histopaque® (Sigma) and 4 × 10^7^ cells were incubated with 500 μl *T. parva* (Muguga sporozoite stabilate P2015/4) for 90 min at 37°C/5% CO_2_. Cells were gently mixed every 15 min during the incubation. After incubation, cells were washed in 15 ml of cRPMI (10% fetal bovine serum, 24 mM of HEPES, 2 mM of L-glutamine and 10 μg/mL of gentamicin) and plated at 2 × 10^6^ cells/well in 24-well cell culture plates. Cells were cultured in cRPMI at 37°C/5% CO_2_ and infection was assessed by intracellular staining the cells with the anti-PIM monoclonal antibody ALS40 (13) followed by flow cytometric analysis. Once established, cell lines were maintained at 37°C/5% CO_2_ and sub-cultured in fresh cRPMI every 2–4 days as needed.

### Bovine IFNγ ELISpot

PBMC from *T. parva*-immune and uninfected control cattle were isolated as described above and exposed to recombinant Tp9 secreted from pEF1α-tPA-Tp9-iresGFP transfected HEK 293T cells. Production of IFNγ by PBMC was measured using ELISpot (MabTech) following the manufacturer's instructions. Briefly, 1 × 10^5^ PBMC were added to MultiScreen ® HTS 96-well plates (Millipore) coated overnight with anti-bovine IFNγ capture antibody (MT17.1, 10 μg/ml). Cells were incubated overnight at 37°C/5% CO_2_ in a humidified incubator with supernatant from HEK 293T cells transfected with pEF1α-tPA-Tp9-iresGFP or pEF1α-GFP. To optimize the amount of recombinant protein to use in the ELISpot assays, a preliminary experiment in which PBMC were exposed overnight to HEK 293T supernatant containing 1, 10, 50, or 100 ng of Tp9 and IFNγ ELISpot performed. Optimal IFNγ production and minimal cell toxicity were obtained using supernatant containing 10 ng of Tp9 (data not shown) and this amount was then used throughout the study. PBMC from immune and control cattle were also stimulated with *T. parva* schizont-infected cell line lysate (20 μl lysate per well, ~1.5 × 10^5^ *T. parva* infected cell equivalents per well) and 20 ng/ml phorbol 12-myristate 13-acetate (Sigma) plus 1 μg/ml Ionomycin (Millipore) as positive controls for *T. parva* specific immune responsiveness and IFNγ production, respectively. After incubation, ELISpot plates were washed five times in PBS and incubated for 2 h with biotinylated anti-bovine IFNγ detection antibody (MT307, 0.25 μg/ml). Plates were again washed five times in PBS, incubated for 1 h with streptavidin-alkaline phosphatase conjugate (1:1,000) (MabTech), washed five more times in PBS and developed using the substrate for ELISpot assay BCIP/NBT-plus (MabTech). ELISpot plates were read and analyzed using an Immunospot® ELISpot reader (Cellular Technology Limited).

### Blockage of MHC Class I and Class II

To investigate the role of MHC class I and class II in IFNγ production, blocking experiments were performed as previously described (31). Briefly, PBMC were isolated as described above and incubated for 15 min at 4°C with a pool of anti-bovine MHC class I and class II monoclonal antibodies (mAb) (1 μg mAb/10^6^ cells) (Supplementary Table 2). Cells were washed once in cRPMI, incubated with recombinant Tp9, and IFNγ ELISpot performed as describe above.

### Cell Depletion

Following PBMC isolation as described above, depletion of CD4, CD8, or γδ T cells was performed to allow better characterization of the Tp9-specific cellular immune response. Monoclonal antibodies to bovine CD4, CD8, TCR δ chain (Supplementary Table 2) were used with MACS® MicroBeads (Miltenyi Biotec) for depletion by following the manufacture's protocol. Briefly, cells were incubated with the monoclonal antibody/ies specific for markers of the cell population(s) to be depleted (1 μg mAb/10^6^ cells) for 15 min at 4°C in MACS buffer (PBS without ${\text{Ca}}_{2}^{+}$ and ${\text{Mg}}_{2}^{+}$, pH 7.0, 0.5% BSA, and 2 mM EDTA). Subsequently, cells were washed twice in MACS buffer and incubated with goat anti-mouse IgG MicroBeads (20 μl MicroBeads/10^7^ cells) (Miltenyi Biotec) for 15 min at 4°C. Cells were then washed twice in MACS buffer and passed through a MACS LS column (Miltenyi Biotec). Efficiency of depletion was evaluated by flow cytometry using the Guava® easyCyte™ HT system (Millipore). CD4 depleted cells, CD8 depleted cells, or CD4/γδ depleted cells were used for IFNγ ELISpot as described above.

## Results

### Tp9 Secretion and Signal Peptide Characterization

**Figure 1:**
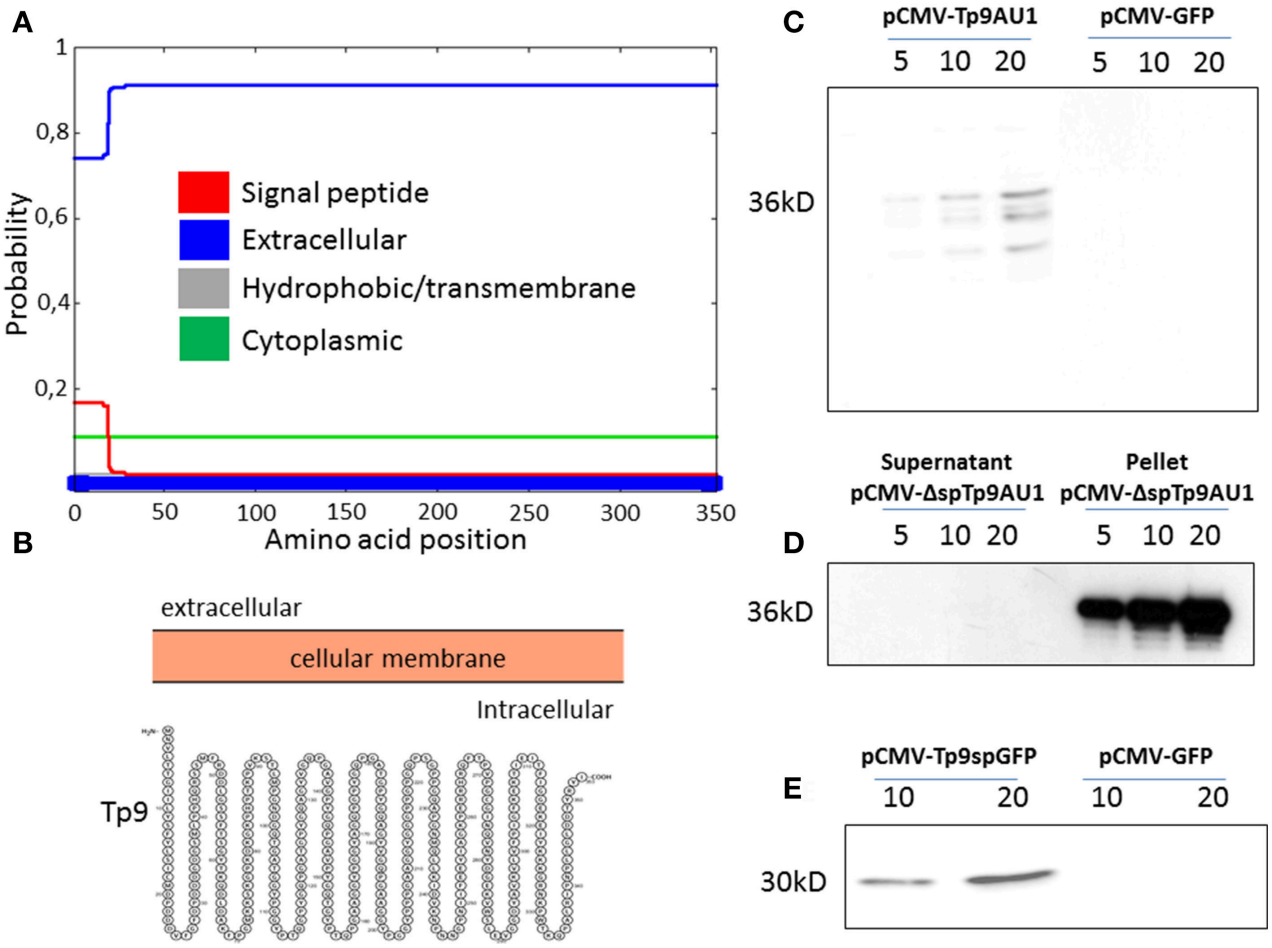
Tp9 expression and secretion by mammalian cells. Server (Phobius) output prediction of transmembrane topology and signal peptides from the amino acid sequence of Tp9 protein **(A)**. A diagram (not on scale) of Tp9 protein subcellular localization as predicted by a different server (Protter) **(B)**. Immunoblot of pCMV-Tp9AU1 transfected HEK 293T cells supernatant and mock pCMV-GFP transfected HEK 293T cells supernatant. Lanes were loaded with different amounts of serum free medium supernatant (5, 10, and 20 μl) at 48 h post transfection. The same number HEK 293T cells were transfected with the same amount of pCMV-Tp9AU1 or pCMV-GFP DNA and with the same efficiency of transfection **(C)**. Immunoblot of serum free medium supernatant and protein extract from pCMV-ΔspTp9AU1 transfected HEK 293T cells. Supernatant lanes were loaded with different amounts of serum free medium supernatant (5, 10, and 20 μl), whereas pellet lanes were loaded with different amounts of protein extract (5, 10, and 20 μg) **(D)**. Immunoblot of pCMV-Tp9spGFP transfected HEK 293T cell supernatant and mock pCMV-GFP transfected HEK 293T cell supernatant. Lanes were loaded with different amounts of serum free medium supernatant (5, 10, and 20 μl) at 48 h post transfection. The same number HEK 293T cells were transfected with the same amount of pCMV-Tp9spGFP or pCMV-GFP DNA and with the same efficiency of transfection **(E)**.

Although no functional data on the secretion of the Tp9 protein have been published to date, it is predicted to be secreted due to the presence of a signal peptide and the lack of a transmembrane domain. *In silico* analysis revealed a putative Tp9 signal peptide that, although highly conserved among Tp9 sequences from different *T. parva* isolates, prompts only weak protein secretion compared to canonical signal peptides of typical mammalian secreted proteins. In fact, according to its amino acid sequence and as predicted by two servers for signal peptide prediction and visualization (Phobius; http://phobius.sbc.su.se/; and Protter; http://wlab.ethz.ch/protter/start/), the signal peptide predictive score of the putative Tp9 signal peptide is low (Figure 1A, red line, score below 0.2; defined cut-off is 0.5), suggesting that the protein is cytoplasmic and not secreted (Figures 1A,B, blue line, score above 0.7; defined cut off is 0.5). However, Tp9 was secreted from HEK 293T cells transfected with pCMV-Tp9AU1, a construct delivering AU1 tagged Tp9 ORF, placed under transcriptional control of the CMV promoter and the bovine growth hormone polyadenylation signal (Figure 1C). Removal of the signal peptide (pCMV-ΔspTp9AU1) completely abrogated Tp9 secretion and the protein was retained inside the cells (Figure 1D). Further, when the Tp9 signal peptide was placed in front of GFP (pCMV-Tp9spGFP), it allowed GFP secretion (Figure 1E). These data corroborate that Tp9 is a secreted protein and possesses a functional signal peptide when it is expressed in mammalian cells.

### Tp9 Signal Peptide Substitution Significantly Increases Protein Secretion

**Figure 2:**
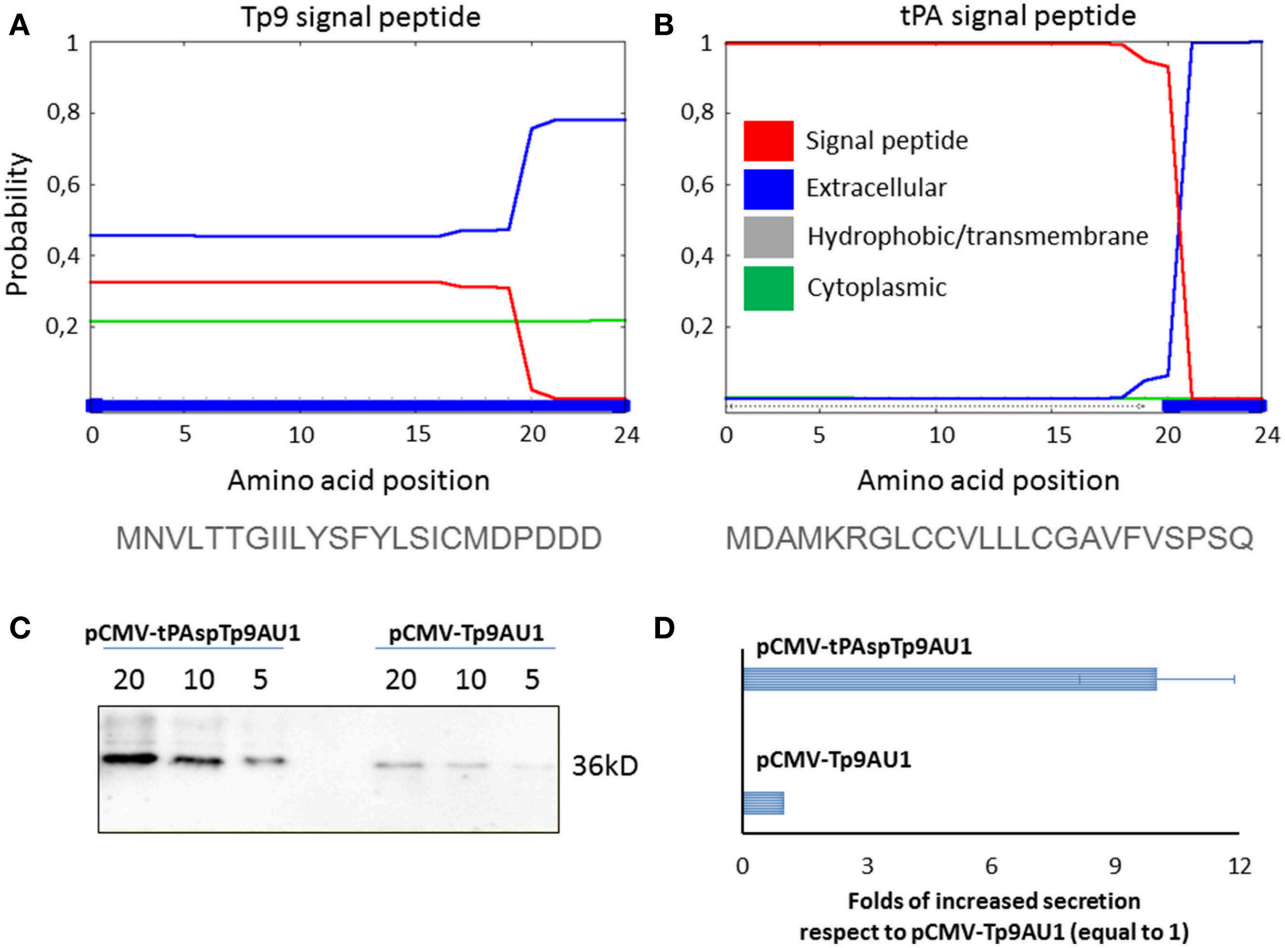
Impact of signal peptide substitution on Tp9 secretion by mammalian cells. Server (Phobius) output prediction of signal peptide from the first 24 amino acid of Tp9 protein sequence **(A)** compared with the first 24 amino acid of human tPA protein sequence **(B)**. Immunoblot of serum free medium containing secreted Tp9 from pCMV-tPAspTp9AU1 or pCMV-Tp9AU1 transfected HEK 293T cells at 48 h post transfection. Each lane was loaded with different amounts of serum free medium supernatant (5, 10, and 20 μl). The same number HEK 293T cells were transfected with the same amount of pCMV-tPAspTp9AU1 or pCMV-Tp9AU1 DNA and with the same efficiency of transfection (100%) **(C)**. Intensity of protein bands in the immunoblot shown in **(C)** was quantified by densitometry (ChemiDoc; MPIMAGING SYSTEM, LAB SOFTWARE, BioRad) and displayed as a bar graph. Data were normalized as a folds of increased secretion, where pCMV-Tp9AU1 signal was considered equal to 1 for each amount of serum free medium loaded **(D)**. The experiment was repeated three times and statistical significance (*p* < 0.001) was assessed by Student's *t*-test.

**Figure 3:**
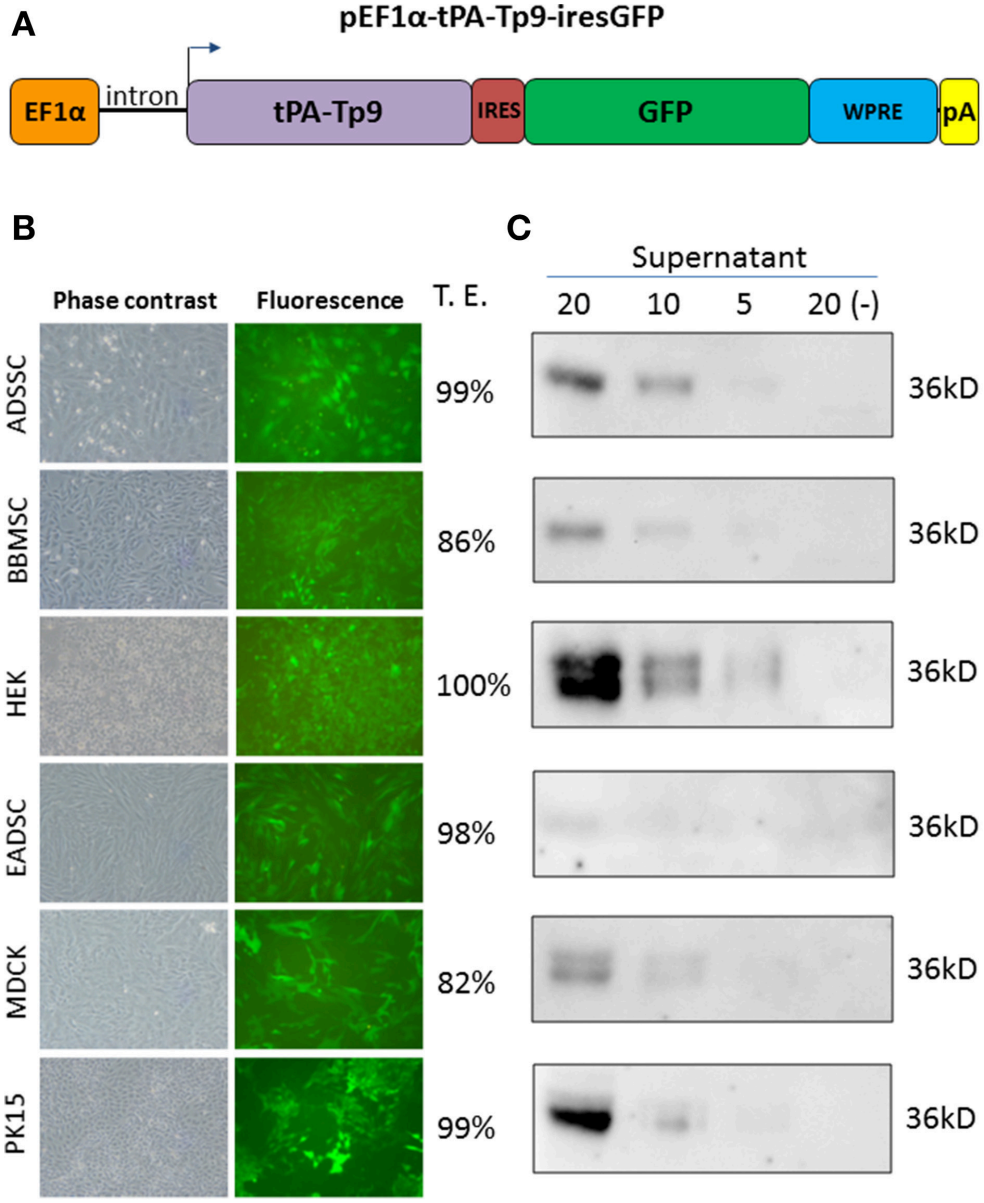
Generation of stably transfected cells secreting Tp9. Diagram of pEF1α-tPA-Tp9-iresGFP lentiviral transfer vector (not on scale) **(A)**. Representative images (10X) of pEF1α-tPA-Tp9-iresGFP transduced ADSSC, BBMSC, HEK 293T, EADSC, MDCK, PK15 cells, and transduction efficiency (T.E.) as measured by cytometry (TALI, Image based cytometer, Invitrogen) **(B)**. Immunoblot of serum-free medium at 48 h post collection from pEF1α-tPA-Tp9-iresGFP stably transduced ADSSC, BBMSC, HEK 293T, EADSC, MDCK, and PK15 cells. Each lane was loaded with different amounts of serum free medium supernatant (5, 10, and 20 μl) **(C)**.

Since Tp9 contains a functional signal peptide, but was only secreted from mammalian cells in small amounts, we sought to determine if the substitution of the native signal peptide with a canonical eukaryotic signal peptide would increase secretion efficiency. Thus, the Tp9 signal peptide was substituted with the human tPA signal peptide, which has a considerably higher signal peptide predictive score than the native Tp9 signal peptide according to the amino acid sequence as predicted by Phobius (http://phobius.sbc.su.se/) (Figures 2A,B). Indeed when HEK 293T cells were transfected with pCMV-tPA-Tp9AU1 (Tp9 containing tPA signal peptide) (Figure 2C), a 10-fold increase in secretion was observed compared to cells transfected with pCMV-Tp9AU1 (Tp9 containing its native signal peptide) (Figure 2D). Further, Tp9 was efficiently secreted from different animal species cells lines transduced with a replication-incompetent lentiviral vector delivering tPA-Tp9AU1 (Figure 3A), as evidenced by efficient GFP production (Figure 3B) and detection of AU1-tagged recombinant Tp9 in cell supernatants by immunoblot (Figure 3C). This lentiviral transfer vector, pEF1α-tPA-Tp9-iresGFP, carries Tp9 and GFP under the transcriptional control of the human elongation factor 1 alfa (EF1α) strong promoter; the GFP ORF was in a bicistronic form with tPAsp-Tp9 through an Internal ribosomal entry site (IRES). After the reconstitution of the replication incompetent lentiviral particles in HEK 293T cells, various cell lines constitutively secreting Tp9 into the medium supernatant were generated as a useful Tp9 antigen source.

### *T. parva*-Immune Cattle Produce Antibodies That Recognize Recombinant Tp9

**Figure 4:**
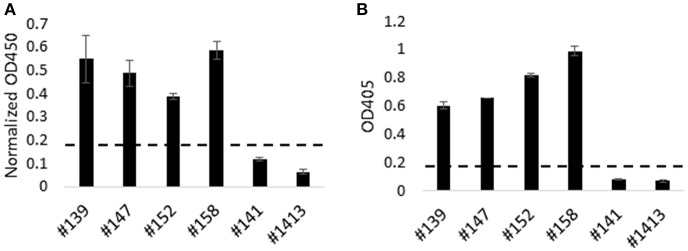
Humoral immune response to Tp9 in *T. parva*-immune animals. *T. parva*-immune cattle (#139, #147, #152, and #158) and uninfected animals (#141 and #1413) were tested by a Tp9 ELISA and *T. parva* PIM ELISA. Presence of antibodies to Tp9 in *T. parva*-immune animals. Results are presented as normalized OD450 (Tp9 OD450—GFP OD450) **(A)**. Results show the presence of anti-PIM antibodies in *T. parva*-immune animals **(B)**. Dashed lines in **(A,B)** indicate 3 standard deviations of the negative samples **(B)**.

Next we utilized HEK-cell-expressed, recombinant Tp9 to determine if *T. parva*-immune cattle produce antibodies to this antigen following infection (Figure 4). HEK cells were chosen for expression due to significantly higher recombinant Tp9 protein secretion from this line compared to other cell lines tested. An ELISA was developed using supernatant of HEK 293T cells containing soluble, full-length, recombinant tPAspTp9 as antigen. Individual wells containing supernatant of HEK 293T cells transfected with a plasmid expressing GFP (pEF1α-GFP) were used as a control to normalize the data. Serum samples from *T. parva*-immune and control cattle were used in the Tp9 ELISA. Results demonstrate the presence of anti-Tp9 IgG antibodies in all four immune animals (Figure 4A). Mean OD values from each of the immune animals were ≥ three standard deviations above the mean OD values from each of the control cattle, indicating the specificity of the assay. Mouse anti-AU1 monoclonal antibody was used to verify binding of Tp9-AU1 to the ELISA plates (data not shown). To confirm *T. parva*-immune status, serum samples from all cattle were also tested in an anti-PIM ELISA as previously described (28) and as expected, the data showed the presence of anti-PIM antibodies in all four infected animals (Figure 4B). Significant anti-Tp9 and anti-PIM antibodies were not detected in serum samples from control cattle.

### Production of IFNγ by PBMC From *T. parva*-Immune Cattle Following *ex vivo* Exposure to Recombinant Tp9

**Figure 5:**
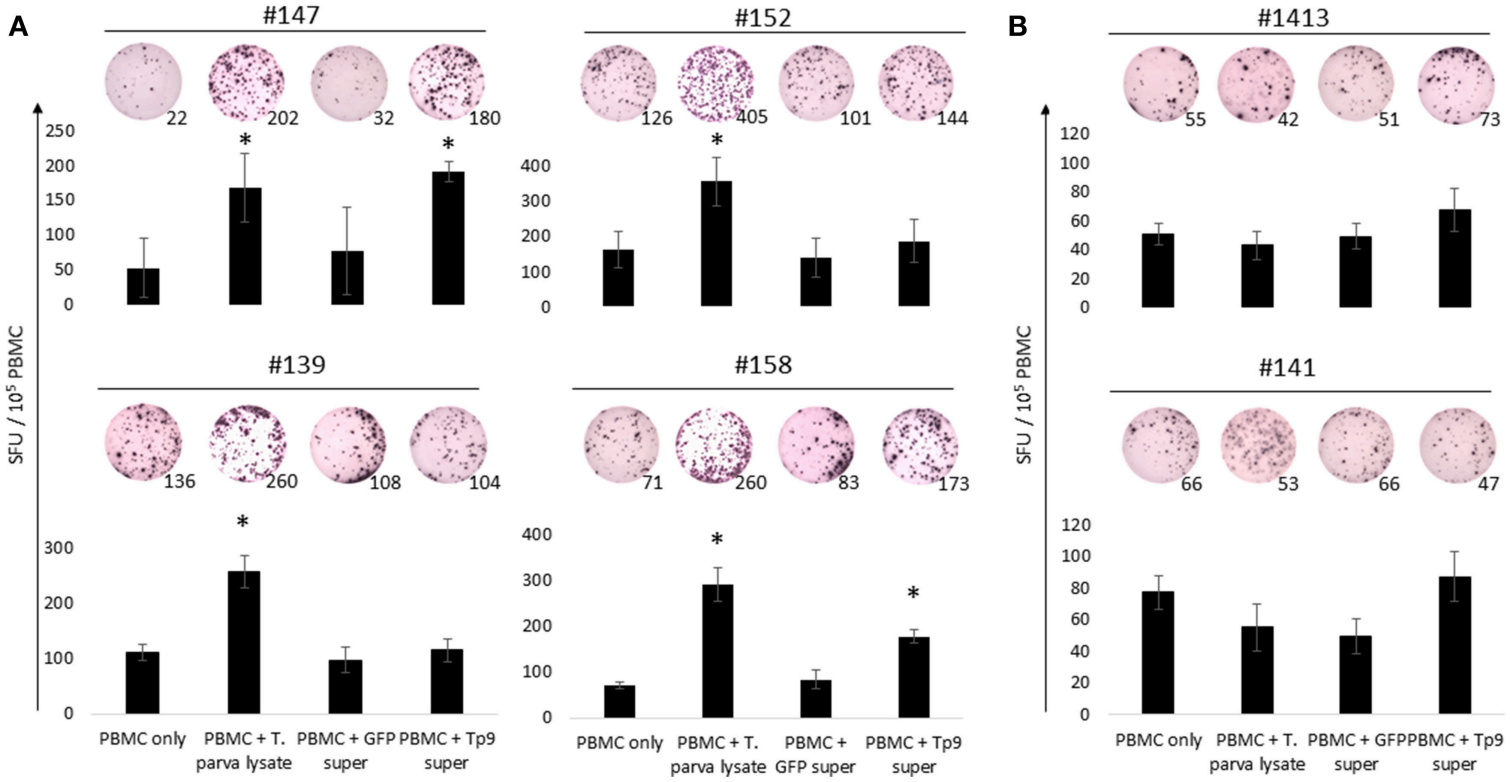
IFNγ production from PBMC exposed to HEK 293T cell supernatant containing Tp9. *T. parva* cell line lysate and supernatant from HEK 293T cells transfected with pCMV-GFP were used as positive and negative controls, respectively. Bar charts show the average of a representative experiment and results are shown as spot forming units (SFU) per 10^5^ PBMC of each animal. A representative ELISpot well is depicted on the top of each bar and numbers represent the total number of IFNγ spots per well. Steers #147, #152, #139, and #158 were *T. parva*-immune animals **(A)**. Steers #1413 and #141 were used as uninfected control animals **(B)**. ^*^p < 0.05.

To further characterize the antigenicity of recombinant Tp9 expressed in mammalian cells, PBMC from *T. parva*-immune cattle were exposed to soluble, secreted Tp9 and IFNγ production evaluated by ELISpot (Figure 5). Significant numbers of PBMC (*p* < 0.5) from two of four immune animals produced IFNγ following exposure to Tp9 compared to cells in medium only or cells exposed to supernatant from pEF1α-GFP transfected HEK 293T cells (Figure 5A). As expected, significant numbers (*p* < 0.5) of PBMC from all four immune cattle produced IFNγ after exposure to *T. parva* cell line lysate (Figure 5A). No significant IFNγ response was detected when PBMC from uninfected animals were exposed to Tp9 or *T. parva* cell line lysate compared to cells in medium only (Figure 5B).

**Figure 6:**
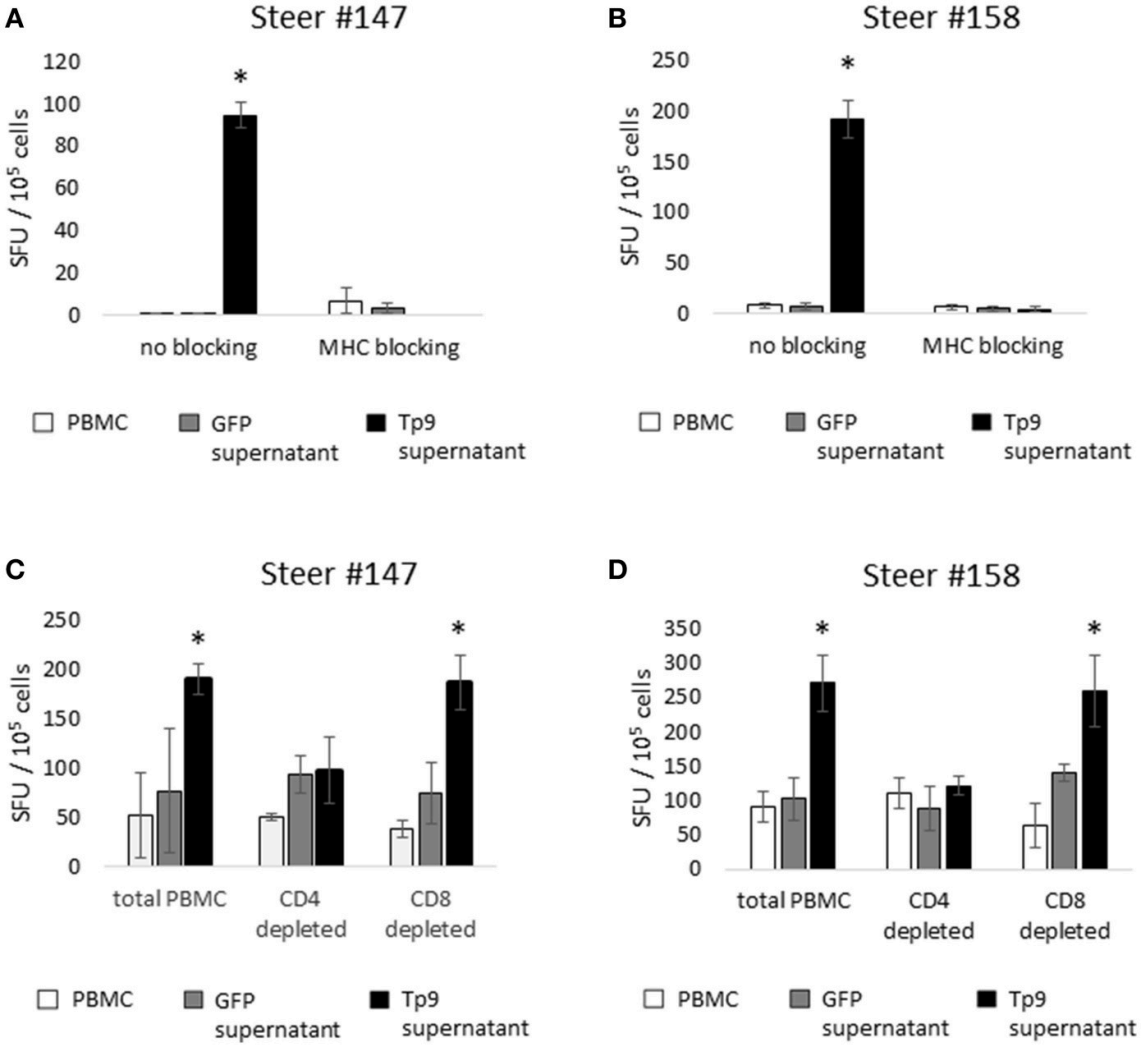
IFNγ production by cells from *T. parva*-immune cattle (steers 147 and 158), as measured by IFNγ ELISpot. Monoclonal antibody blockade of MHC class I and class II molecules abrogates the IFNγ response to Tp9 in both *T. parva*-immune steers **(A,B)**, indicating that the response to Tp9 is MHC-restricted. IFNγ production by total PBMC, the CD4+ T-cell depleted cell population, and the CD8+ T-cell depleted cell population from *T. parva*-immune steers 147 **(C)** and 158 **(D)** stimulated overnight with recombinant Tp9. In both steers, significant responses are observed only in the total PBMC and CD8+ T-cell depleted populations, suggesting that CD4+ T cells are the primary source of IFNγ production in *T. parva*-immune cattle. In all panels, results are shown as spot forming units (SFU) per 10^5^ PBMC. ELISpot experiments were repeated 3 times, and samples tested in triplicate each time. ^*^p < 0.05.

Next we investigated potential mechanisms to explain the variability of IFNγ production by PBMC from the *T. parva*-immune cattle following exposure to recombinant Tp9. By genotyping MHC class I and class II loci we found that steer #147, one of the animals whose cells produced IFNγ in response to Tp9, was homozygous A14/A14 for MHC class I and heterozygous 0902A/1401B for MHC class II. Steer #158, the other animal whose cells produced IFNγ in response to Tp9, was heterozygous for both MHC class I and class II, A10/A19 and 1001A/0101A, respectively (Table 1). The two *T. parva*-immune steers whose cells did not produce IFNγ following exposure to Tp9 were heterozygous for both MHC class I and class II. Steer #152 was A11/A14 and 0101A/1101A for MHC class I and class II, respectively, and animal #139 was A10/A12 and 1001A/1501A for MHC class I and class II, respectively (Table 1). When the IFNγ ELISpot was repeated in the presence of a pool of anti-bovine MHC class I and class II monoclonal antibodies, IFNγ response was completely abrogated in steers #147 and #158 (Figures 6A,B). Collectively, these results demonstrate that PBMC from *T. parva*-immune cattle produce IFNγ following *ex vivo* exposure to recombinant, mammalian-expressed Tp9, consistent with anti-Tp9 cellular immune response development during natural infection. Furthermore, these data also confirm that the cellular immune response to Tp9 is MHC-restricted and dependent on MHC genotypes.

**Table 1:** Genotype of MHC class I and class II of the *Theileria parva*-immune animals used in this study.

**Animal ID**	**MHC I**	**MHC II**
#147	A14/A14	0902A/1401B
#152	A11/A14	0101A/1101A
#139	A10/A12	1001A/1501A
#158	A10/A19	1001A/0101A

### CD4^+^ T Lymphocytes Are the Major Source of IFNγ Following *ex vivo* Exposure to Recombinant Tp9

We next sought to elucidate which lymphocyte population was the primary source of IFNγ production following *ex vivo* exposure to recombinant, mammalian expressed Tp9. In cattle, IFNγ is primarily produced by CD4^+^ T cells, CD8^+^ T cells, and γδ T cells in a MHC-restricted fashion, and to a lesser extent by NK cells in a non-MHC-restricted manner (32–34). Therefore, we performed depletion experiments followed by ELISpot to determine which cell population(s) produce(s) IFNγ in response to Tp9. MACS MicroBeads depletion of CD4^+^, CD8^+^, or CD4^+^ and γδ T cells resulted in elimination of more than 90% of the targeted population (data not shown). In steers #147 and #158, PBMC populations depleted of CD8^+^ T cells and stimulated overnight with recombinant Tp9 contained similar numbers of IFNγ-producing cells as the total PBMC population (Figures 6C,D). In steers #139 and #152, there was no significant CD4^+^ T-cell IFNγ response to Tp9 (data not shown). In all four steers, significant IFNγ-producing cells were not detected in PBMC depleted of CD4^+^ T cells or CD4^+^ T cells and γδ T cells compared total PBMC and cells stimulated with negative control antigen (supernatant from HEK 293T cells transfected with pEF1αGFP). Taken together, these results strongly suggest that CD4^+^ T cells are the major source of IFNγ following overnight exposure to Tp9 (Figures 6C,D).

## Discussion

In the present study we performed a molecular characterization of *Theileria parva* antigen Tp9 and used a recombinant, mammalian expressed Tp9 to study its antigenic properties in cattle. Initially, we showed that native sequence of Tp9 presents a functional, but atypical signal peptide. Interestingly, secretion of Tp9 from mammalian cells was significantly increased by replacing the native secretion signal by a canonical eukaryotic signal peptide. Using this recombinant, full-length Tp9 secreted from mammalian cells, we showed that *T. parva*-immune cattle develop both humoral and cellular immune response to this antigen. Our results also indicate that CD4^+^ T cells are the major source of IFNγ following *ex vivo* exposure to recombinant, mammalian-expressed Tp9.

ECF, the acute lymphoproliferative form of *T. parva* infection, has a major economic impact on cattle production in endemic areas of Eastern, Central, and Southern Africa (1, 35). Cattle can be protected against ECF by ITM vaccination, where animals are inoculated with live *T. parva* sporozoites and simultaneously treated with a long-acting oxytetracycline (7, 29). ITM-vaccinated animals usually undergo an asymptomatic infection, and develop long-lasting immunity to similar strains of *T. parva*. In addition to ITM, cattle in endemic areas can also be treated with acaricides to decrease infestation by the *Theileria parva* tick vector, *Rhipicephalus appendiculatus*. Both adoption of ITM vaccination and acaricide use are curtailed by numerous financial, logistical and environmental drawbacks and therefore, there is an urgent need for the development of affordable, next-generation vaccines to control *T. parva* (35). To achieve this goal, it is critical to discover and characterize new *T. parva* candidate vaccine antigens and to optimize suitable systems for recombinant antigen expression and delivery. In the present study, we expressed and secreted full-length Tp9, a recently-discovered, candidate *T. parva* vaccine antigen, using a mammalian system and characterized its antigenic properties in cattle.

Previous attempts to express recombinant *T. parva* proteins in prokaryotic or eukaryotic systems for vaccine purposes have been challenging and the process has been hindered due to protein insolubility and misfolding, among other issues (15, 16, 20). Recently, we were able to express full-length *T. parva* p67 antigen in mammalian cells and demonstrated that the recombinant antigen retained its immunogenic properties (21). Considering the technical and biological challenges associated with the expression of *Theileria* sp. antigens, it is desirable to develop a practical, systematic eukaryotic system to express potential vaccine target antigens. In the present study, we efficiently expressed full-length Tp9 in several mammalian cell lines by either transfecting cells with pCMV-tPA-Tp9AU1 (Tp9 containing tPA signal peptide) or transducing cells with a replication-incompetent lentiviral vector delivering tPA-Tp9AU1. By cloning the tPA signal peptide upstream of the Tp9 gene, we were able to express and obtain efficient secretion of the Tp9 antigen from all mammalian cell lines tested. The secreted antigen was subsequently used for antigenic assessment. These results demonstrate the plasticity and usefulness of the presented expression system to produce antigens that can be further tested in *T. parva* vaccine formulations.

Molecular analysis performed in this study demonstrated that Tp9 contains a unique signal peptide with only moderate activity in mammalian cells. By replacing the native Tp9 signal peptide with the human tPA signal peptide, we achieved a 10-fold increase in secretion of Tp9 from HEK cells. Apicomplexan parasites have evolved specific molecular mechanisms to adapt to their respective intracellular niches and interact with their hosts (36). One of these mechanisms is the secretion and trafficking of parasite proteins into the parasitophorous vacuole and subsequently into host cell cytoplasm (37). However, in contrast to other Apicomplexan parasites, such as *Toxoplasma, Cryptosporidium*, and *Plasmodium, T. parva* does not form a parasitophorous vacuole and resides free into the cytoplasm of infected lymphocytes (38). A recent study demonstrated that the *T. annulata* antigen Ta9, an ortholog of Tp9, does indeed localize to the host cell cytoplasm, where it is involved in the activation of the transcription factor AP-1, which leads to the expression of cell proliferation genes (39). Here, we demonstrated that the Tp9 signal peptide is only weakly functional to achieve secretion in mammalian cells. However, it is likely that this signal peptide functions effectively within the parasite to achieve secretion of Tp9 into the cytoplasm of infected lymphocytes, where it may interact with host signaling pathways.

As we demonstrated that transiently transfected cells secreted Tp9 in the cell culture medium allowing the recovery of the protein directly from cell supernatant, it was of interest to generate stable cell lines constitutively secreting Tp9, as a source of protein for various purposes. To achieve this, a third generation, replication incompetent lentiviral vector delivering tPAsp-Tp9 expression cassette was generated. After lentiviral particles reconstitution, several cell lines from different animal species, were transduced with an efficiency ranging of 86–100%, measuring GFP expression by Tali assay. The level of GFP expression in the tPAsp-Tp9AU1 lentiviral transduced cells reflects the expression of Tp9 protein because Tp9 ORF is located upstream of the IRES sequence in a bi-cistronic form with the GFP ORF. Secreted Tp9 can be potentially purified from serum-free medium of transduced cells and used for immunization and immunological assays, without the need of further purification. The bi-cistronically expressed GFP enables positive cells to be sorted to increase the number of Tp9 secreting cells.

Two out of four *T. parva*-immune cattle in our study had significant *ex vivo* cellular immune responses to Tp9, consistent with development of an anti-Tp9 T-cell response during infection. Numerous studies have shown that T cells from *T. parva*-immune cattle produce IFNγ in response to *T. parva*-infected cells and to a subset of *T. parva* antigens (10, 40, 41). Immune protection against *T. parva* requires the development of robust MHC class I and class II restricted T-cell responses, and it is likely that efficient induction and recall of such CD8 T cell responses also requires CD4 T cell responses. Outbred cattle populations express diverse MHC genotypes (42–45), and the antigenic specificities of T cells against *T. parva* antigens vary between cattle of different MHC genotypes, as demonstrated by our data on Tp9. MHC class I and II antibody blockage abrogated the IFNγ response in both animals, confirming that the response is MHC-restricted. This indicates that a subset of the MHC class I and class II molecules expressed by these cattle present Tp9 epitopes that are subsequently recognized by T cells during T-cell response priming in acute infection.

Not only did 2/4 *T. parva*-immune cattle in this study exhibit a cellular immune response to Tp9, but all four animals, representing various MHC class I and class II genotypes, had a significant anti-Tp9 antibody response. Tp antigens, including Tp9, were identified by screening CD8^+^ T cell lines from *T. parva*- immune cattle (10, 18), and Tp9 was initially identified as a *T. parva* schizont-antigen. A recent proteomic study reported that Tp9 is also expressed by *T. parva* sporozoites (46). Considering that sporozoites are the *T. parva* stage that is infectious to cattle, these results have implications for vaccine development. After inoculation by ticks, sporozoites are only briefly exposed to fluids, including immunoglobulins, prior to entering bovine lymphocytes. It is likely that B cells are exposed to Tp9 within the milieu of lysed and necrotic schizont-infected cells found in various tissues later in disease. Although further studies are required to localize Tp9 within both *T. parva* sporozoites and schizont-infected lymphocytes, the fact that an anti-Tp9 antibody response is generated during *T. parva* infection in cattle of various MHC class I and II genotypes provides rationale for further testing of the Tp9 antigen as a component of a multivalent subunit vaccine for *T. parva*.

Development of protective, affordable vaccines is the most cost-effective approach to control both animal and human infectious diseases, especially those neglected conditions that primarily affect people in developing regions of the world (47–49). A majority of the cost associated with ECF morbidity and mortality is borne by smallholder pastoralist farmers (7) in sub-Sahara Africa. ITM, the most effective means of ECF prevention, is expensive and logistically challenging. Therefore, development of new vaccines to control ECF is urgently needed to mitigate the effects of the disease. Here, we investigated the molecular and antigenic properties of Tp9, a *T. parva* candidate vaccine antigen expressed by sporozoite and schizont parasite stages. Data show that *T. parva*-immune animals develop both humoral and cellular immune responses to Tp9 during infection. The demonstrated antigenicity of Tp9 provides rationale for further evaluation of the effectiveness of this antigen as a component of a subunit vaccine to control *T. parva*.

## Ethics Statement

This study was carried out in accordance with the recommendations of The U.S. Animal Welfare Act (United States Code, Title 7, Chapter 54, sections 2131–2159) and Animal Welfare Regulations (Code of Federal Regulations, Title 9, Chapter 1, Subchapter A, parts 1–4). The protocol was approved by the Washington State University Institutional Animal Care and Use Committee, protocol number 4980.

## Author Contributions

RB, DK, GD, and LF conceived and designed the experiments. RB, VF, GT, GD, and LF performed the experiments. RB, VF, GT, GD, DK, and LF analyzed the data. RB, TC, WM, DK, GD, and LF contributed with reagents, materials, analysis tools. RB, GT, LF, VF, and GD wrote the paper. All authors read and approved the final manuscript.

### Conflict of Interest Statement

The authors declare that the research was conducted in the absence of any commercial or financial relationships that could be construed as a potential conflict of interest.
