# Supplementary figures and images for: Molecular and Antigenic Properties of Mammalian Cell-Expressed Theileria parva Antigen Tp9
Source: Front Immunol. 2019 Apr 29;10:897. doi: 10.3389/fimmu.2019.00897 (PMC6501543; doi:10.3389/fimmu.2019.00897)

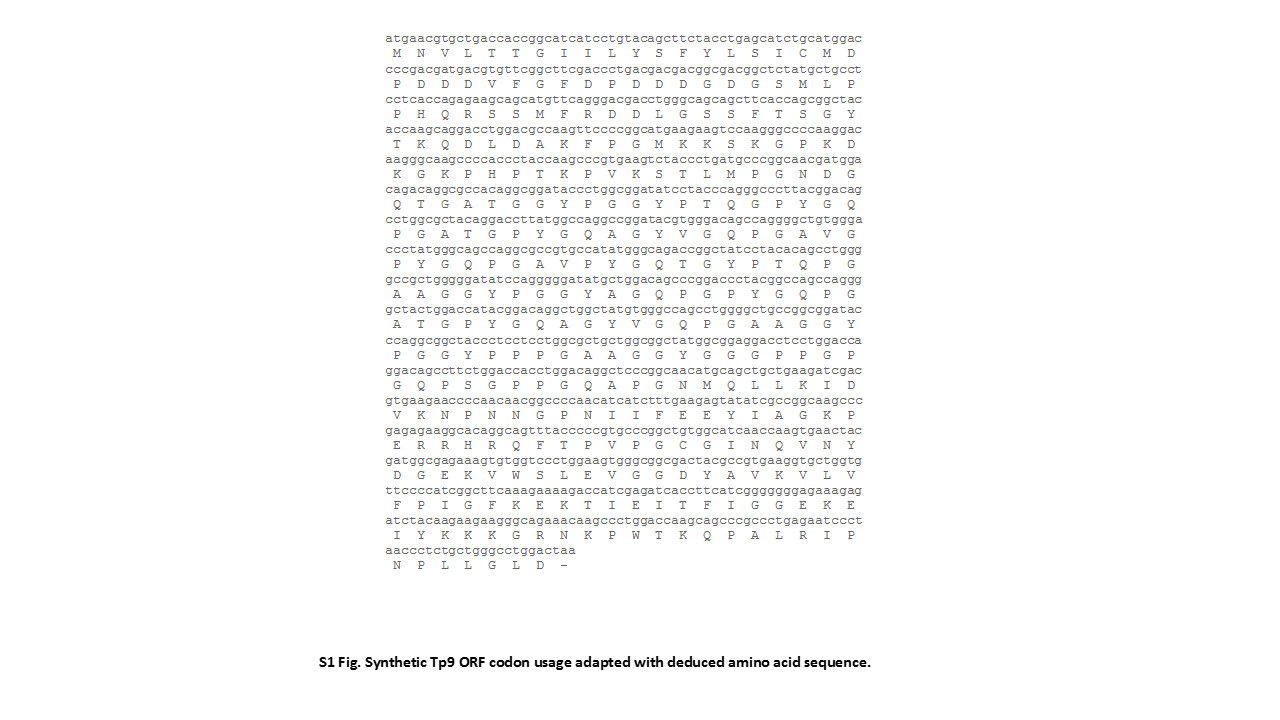

Supplement: Supplementary file 1 [file Image_1.TIF]
