# Supplementary material for: Molecular and Antigenic Properties of Mammalian Cell-Expressed Theileria parva Antigen Tp9
Source: Front Immunol. 2019 Apr 29;10:897. doi: 10.3389/fimmu.2019.00897 (PMC6501543; doi:10.3389/fimmu.2019.00897)
Supplement: Supplementary file 2 [file Table_1.pdf]

Supplementary Table 1. List of primers used in this study.

Primer name	5'-3' Primer sequence
Tp9 sense	ccccgctagcccacc atg cag gaa atc cac gcc agg ttc agg
Tp9 AU1 antisense	cccccccggg tta gat gta ccg gta ggt gtc gtc cag gcc cag cag agg gtt agg
*Sma*I-*Nhe*I Tp9 sense	cccccccggggctagcccacc atg gac gct atg gac gct atg aag agg ggc ctg tgc
Tp9SP-Tp9 sense	cccccccggggctagcccacc atg aac gtg ctg acc acc ggc atc atc ctg tac agc ttc tac ctg agc atc tgc atg gac ccc gac gat gac gtg ttc
p9SP-GFP sense	cccgctagcccacc atg aac gtg ctg acc acc ggc atc atc ctg tac agc ttc tac ctg agc atc tgc atg gac ccc gac gat gac gtg agc aag ggc gag gag ctg ttc acc
GFP-AU1 antisense	ccccctcgag tta gat gta ccg gta ggt gtc ctt gta cag ctc gtc cat gcc gag
