# Supplementary material for: Molecular and Antigenic Properties of Mammalian Cell-Expressed Theileria parva Antigen Tp9
Source: Front Immunol. 2019 Apr 29;10:897. doi: 10.3389/fimmu.2019.00897 (PMC6501543; doi:10.3389/fimmu.2019.00897)
Supplement: Supplementary file 3 [file Table_2.pdf]

Supplementary Table 2. List of monoclonal antibodies used for cell depletion and MHC blocking experiments.

monoclonal antibody	isotype	specificity	source
H58A	IgG2a	MHCI	WSU Monoclonal Antibody Center
PT85A	IgG2a	MHCI	WSU Monoclonal Antibody Center
TH14A	IgG2a	MHCII BoLA DR	WSU Monoclonal Antibody Center
TH81A5	IgG2a	MHCII BoLA DQ	WSU Monoclonal Antibody Center
CAT82A	IgG1	MHCII	WSU Monoclonal Antibody Center
7C2B	IgG2a	CD8α	WSU Monoclonal Antibody Center
ILA11A	IgG2a	CD4	WSU Monoclonal Antibody Center
GB21A	IgG2b	TCR1 δ chain	WSU Monoclonal Antibody Center
